# Investigation of Neuropsychopharmacological Effects of a Polyherbal Formulation on the Learning and Memory Process in Rats

**DOI:** 10.4103/0975-1483.80296

**Published:** 2011

**Authors:** JS Shah, RK Goyal

**Affiliations:** *Department of Pharmacology, Shri Sarvajanik Pharmacy College, Mehsana, India*; 1 *The Maharaja Sayajirao University of Baroda, Vadaodara, Gujarat, India*

**Keywords:** Elevated plus maze, passive avoidance learning, short-term memory, transfer latency

## Abstract

**Objective:**

To investigate the neuropsychopharmacological effect of a polyherbal formulation (PHF) on the learning and memory processes in rats.

**Materials and Methods:**

PHF contains *Withania somnifera (Ashwagandha), Nardostachys jatamansi (Jatamansi), Rauwolfia serpentina (Sarpagandha), Evolvulus alsinoides (Shankhpushpi), Asparagus racemosus (Shatavari), Emblica officinalis (Amalki), Mucuna pruriens (Kauch bij extract), Hyoscyamus niger (Khurasani Ajmo), Mineral resin (Shilajit), Pearl (Mukta Shukhti* Pishti), and *coral calcium (Praval pishti)*. Its effect (500 mg / kg, p.o.) on the learning and memory processes was tested. The activity of PHF on memory acquisition and retention was studied using passive avoidance learning and elevated plus maze model (EPM) in rats.

**Results:**

The animals treated with PHF showed a significant decrease in transfer latency as compared to the control group in EPM. PHF also produced significant improvement in passive avoidance acquisition and memory retrieval, as compared to the controls and reduced the latency to reach the shock free zone (SFZ) after 24 hours.

**Conclusion:**

The PHF produces significant improvement in passive avoidance acquisition and memory retrieval in rats, which needs further investigation.

## INTRODUCTION

With the increasing number of elderly people in the world population, the need for drugs to treat cognitive disorders, such as senile dementia and Alzheimer’s disease, have acquired special urgency. There is a paucity of modern drugs / agents facilitating acquisition, retention, and retrieval of information and knowledge. Ayurveda claims that several plants, the so-called ‘*medhya*’ plants, possess such activities. Scientific literature is continuously reporting plant drugs having neuromodulatory activity. *Shankhapushpi* (leaf) is one of the prime *medhya* plants of Ayurveda, which may be useful for neural regeneration and synaptic plasticity. *Jataamansi* (rhizome) appears to be an excellent candidate for a potential inhibitor of acetylcholinesterase (AChE). *Ashwagandha* (root) is another important anti-aging plant.[1]

The polyherbal formulation contains *Withania somnifera (Ashwagandha), Nardostachys jatamansi* (Jatamansi), *Rauwolfia serpentina (Sarpagandha), Evolvulus alsinoides (Shankhpushpi), Asparagus racemosus (Shatavari), Emblica officinalis (Amalki), Mucuna pruriens (Kauch bij extract), Hyoscyamus niger (Khurasani Ajmo)*, Mineral resin (*Shilajit*), Pearl (*Mukta Shukhti Pishti*) and coral calcium (*praval pishti*). This formulation has been known for its anti-anxiety, anti-stress, adaptogenic, and memory enhancing activity.[2] *Withania somnifera, Emblica officinalis, Asparagus racemosus* and *shilajit* are classified in Ayurveda as *rasayanas*, which are reputed to promote physical and mental health, improve the defense mechanisms of the body, and enhance longevity. These attributes are similar to the modern concept of adaptogenic agents, which are known to provide protection to the human physiological system against diverse stressors.[3] *Evolvulus alsinoids* has been used traditionally as a brain tonic, sedative, anthelmintic, antiepileptic, and against leukoderma.[4] Seeds of *M. pruriens* also possess antioxidant, hypoglycemic, lipid lowering, and neuroprotective activities.[5] *Nardostachys jatamansi* helps to promote physical and mental health, augment resistance of the body against disease, and has shown potent antioxidant activity. It also shows a marked tranquilizing activity, as well as, hypotensive, hypolipidemic anti-ischemic, anti-arrythmic, hepatoprotective, anticonvulsant, and neuroprotective activities.[6]

There is a lack of scientific data regarding the effect of PHF on CNS. The present study is, therefore, focused on the evaluation of the neuropsychopharmacological activity of PHF in rats.

## MATERIALS AND METHODS

### Animals

Albino rats weighing between 200 and 250 g were kept in groups of four and housed in polypropylene cages, in air-conditioned rooms (25 – 28° C), under a standard light / dark cycle. The rats were fed with a standard pellet diet (Pranav Agro Seeds, Vadodara) and water *ad libitum*. The animals were kept for seven days in a laboratory for habituation. The animals were maintained in standard environmental conditions and fed with standard diet and water. The animals were divided into four groups: Control (10 ml/kg saline, orally), Piracetam treated (100 mg/kg), Diazepam treated (2.0 mg/kg).

### Ethical clearance

Allstudies were carried out in accordance with the guidelines provided by the Indian Council for Medical Research and the Committee for the Purpose of Control and Supervision of Experiments on Animals (CPCSEA), New Delhi, India. Moreover, the Institutional Animal Ethical Committee of the Shri Sarvajanik Pharmacy College, Mehsana, approved the study.

### Drugs

Piracetam was received as a gift sample from Torrent Pharmaceuticals Ltd., Indrad, Gujarat, India, The Polyherbal Formulation (PHF) was received as a gift sample from Tonix Healthcare, Ahmedabad, India. Diazepam (Calmpose) was purchased from the local market. PHF contains *Withania somnifera (Ashwagandha), Nardostachys jatamansi (Jatamansi)*, Rauwolfia serpentina (Sarpagandha), Evolvulus alsinoides (Shankhpushpi), *Asparagus racemosus (Shatavari), Emblica officinalis (Amalki), Mucuna pruriens (Kauch bij extract), Hyoscyamus niger (Khurasani Ajmo)*, Mineral resin (*Shilajit*), Pearl (*Mukta Shukhti Pishti*), and coral calcium (*praval pishti*). PHF powder was suspended in distilled water and administered orally.

### Preliminary acute toxicity test

Healthy adult male Wistar rats (150 – 200 g) were subjected to acute toxicity studies as per the guidelines (AOT 425) suggested by the Organization for Economic Cooperation and Development (OECD-2000). The rats were observed continuously for eight hours, for behavioral and autonomic profiles and for any sign of toxicity or mortality, up to a period of 14 days.[7]

### General pharmacological observation

Behavioral effects of the PHF extract (500 mg / kg) were assessed by the method described by Irwin *et al*., (1968). The rats were treated with PHF extract 500 mg / kg. The animals were then placed in an observation cage and observed after 30 minutes of administration up to two hours, for behavioral changes. The observation parameters consisted of body position, locomotion, rearing, righting reflex, lacrimation, alertness, and reactivity to touch stimuli.[8]

### Effect on locomotor activity

Locomotor activity was recorded by using a digital activity cage (Actophotometer Space-lab, India). Three groups of animals were used (n = 6). Each rat was individually placed in the actophotometer for five minutes. Two groups of animals were orally treated with the saline and PHF extract (500 mg/kg). After 60 minutes the rats were placed individually in the actophotometer for recording the basal activity score. Group III receivedreference standard diazepamat a dose of 2.0 mg / kg (i.p.) 30 minutes before the test. Mean change in the locomotor activity was recorded for each group.[9]

### Passive avoidance learning[10]

The apparatus consisted of an electric grid (35 × 35 × 12 cm) with a wooden piece (8 × 5 × 1 inches) as a shock free zone (SFZ) in the center and the entire grid had a perplexed enclosure. Each rat was placed individually on the electric grid and was allowed to explore the apparatus for about one minute. The start button was pressed after selecting the proper voltage of current (20 V) for foot shock. The start button was kept pressed till the animal reached the centrally located shock free zone (SFZ). The latency (in seconds) and number of mistakes that the animal made in 15 minutes was noted. The animal that took more than two minutes to reach the SFZ was rejected. The animal was put back on the electric grid and the procedure was repeated to get at least three basal readings so that the animal got acquainted with the task (trained). The latency of the control animals in reaching the SFZ and the number of mistakes in 15 minutes were compared with those of the treated animals.

### Elevated plus-maze test[11]

The elevated plus-maze consisted of two open (50 × 10 cm) and two closed arms (50 × 10 × 40 cm) for rats facing each other, with an open roof. A fine white line was drawn in the middle of the floor of each enclosed arm. The entire maze was elevated at a height of 50 cm for the rats. The animals were placed individually at the end of either of the open arms and the time the animal took to move from the open to the enclosed arm (transfer latency) was noted on the first day. Transfer latency (TL) was the elapsed time between the time the animal is placed in the open arm and the time at which all its legs have crossed the white line, into the enclosed arm. The animal was allowed to move freely to explore the apparatus for at least 20 seconds. The drugs were administered at least 15 – 30 minutes prior to the first trial. The transfer latency was again recorded 24 hours later. If the animals did not enter the enclosed arm within 60 seconds on the second trial, TL was assigned as 60 seconds. Transfer latency measured on the first and second days served as parameters for acquisition and retrieval, respectively. Each animal was used only once.

### Statistical analysis

The observations are reported as mean ± SEM. The statistical analysis was carried out using two-tailed Student’s paired t-test. *P* values < 0.05 were considered as significant.

## RESULTS

### Preliminary acute toxicity test

All the rats were free of any toxicity up to the dose of 2 g / kg, without any mortality. From this data, the 500 mg / kg dose of PHF was selected for further study.

### General pharmacological observation

The rats that were orally treated with the PHF extract (500 mg / kg) and submitted to the general observations did not show any difference in their behavior and parameters determined during the observation periods. They were alert, with normal grooming, touch response, and pain response. Alertness, limb tone, and grip strength were normal and the animals did not show staggering gait or contractions.

### Effect on locomotor activity

The PHF (500 mg / kg) did not produce any significant reduction in locomotor activity when compared with the control animals. However, the diazepam-treated group showed a statistically significant decrease in locomotor activity when compared with the control group [Table 1].

**Table 1:** Effect of PHF and Diazepam on locomotor activity in rats

Treatment	Mean change in locomotor activity
Saline (1 ml / kg)	25.8 ± 3.14
PHF (500 mg / kg)	30.5 ± 5.25
Diazepam (2 mg / kg)	80.7 ± 3.82*

n = 6, Values are each in mean ± S.E.M.

* indicates *P* < 0.05 (Two tailed Student’s paired t-test).

### Passive avoidance learning and memory

**Figure 1:**
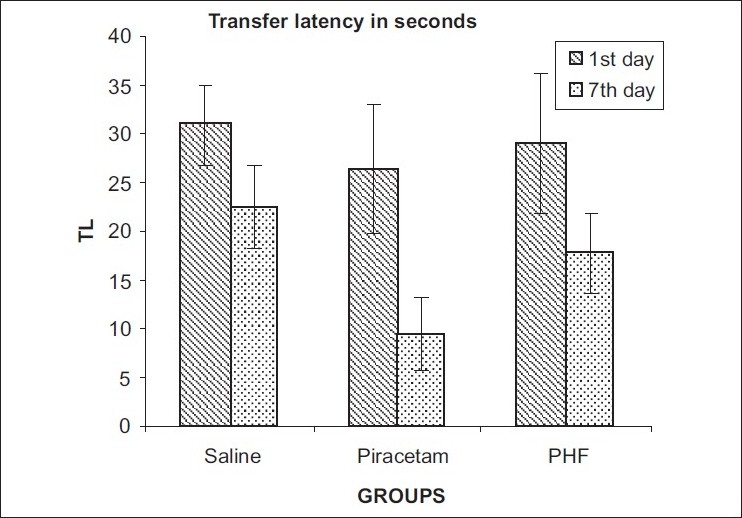
Effect on transfer latency at the end of 24 hours in passive avoidance learning and memory model in rats

**Figure 2:**
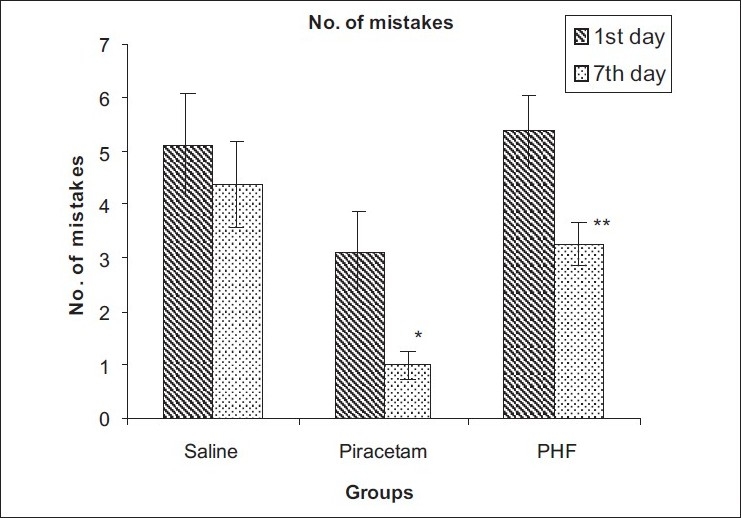
Effect on the number of mistakes in 15 minutes in passive avoidance learning and memory model in rats

The transfer latency to reach the shock free zone on the seventh day was not significantly changed in both the treatment groups as compared to the first day. However, the number of mistakes in 15 minutes were significantly decreased in the Piracetam-treated group (from 3.125 ± 0.76 to 1.0 ± 0.26, *P* < 0.05) and the PHF-treated group (from 5.37 ± 0.67 to 3.25 ± 0.41, *P* < 0.01) at the seventh-day interval. Similarly transfer latency for acquisition and retention was not significantly altered in both the treatment groups at the end of 24 hours [Tables 2 and 3 Figures 1 and 2].

**Table 2:** Effect on passive avoidance learning and memory effects in rats

Treatment (mg / kg)	n	TL+ in seconds	No. of mistakes in 15 minutes (mean ± SEM)
Saline, 1 ml, first day	6	30.87 ± 4.12	5.12 ± 0.97
Saline, 1 ml, seventh day	6	22.37 ± 4.35	4.37 ± 0.82
Piracetam, 100, first day	6	26.37 ± 6.58	3.12 ± 0.76
Piracetam, 100, seventh day	6	9.37 ± 3.79	1.0 ± 0.26*
PHF, 500, first day	6	29 ± 7.25	5.37 ± 0.67
PHF, 500, seventh day	6	17.66 ± 4.03	3.25 ± 0.41**

+ TL: Latency to reach shock free zone, Values are each in mean ± S.E.M.

* indicates *P* < 0.05,

** indicates *P* < 0.01, when compared to day one values (Two tailed Student’s paired t-test

**Table 3:** Effect on transfer latency in passive avoidance requisition and retention model in rats

Treatment (mg / kg)	n	Transfer latency (in seconds) measured after	
		1 hour	24 hours
Saline	6	30.87 ± 4.12	26.75 ± 4.58
Piracetam, 100	6	26.37 ± 6.58	23.62 ± 5.53
PHF, 500	6	29 ± 7.25	26.33 ± 6.82

### Elevated plus maze

There was no significant change in the number of preferences for the open arm in all the three groups between the first day and seventh day interval. The number of open arm entries were not significantly changed in the saline-treated and Piracetam-treated group, but a significant fall from 4.5 ± 0.55 to 2.25 ± 0.52 was observed in the PHF-treated group (*P* < 0.05) [Table 4].

Time spent in the open arm in five minutes was not significantly changed at the end of the seventh day in the treatment groups, whereas, the total number of entries decreased significantly in the Piracetam-treated group (from 41.75 ± 4.5 to 19.25 ± 2.01, *P* < 0.001) and in the PHF-treated group (from 31 ± 3.13 to 14.87 ± 0.9) *P* < 0.001) at the end of the seventh day [Table 4].

**Table 4:** Effect of Polyherbal formulation on elevated plus maze model in Rats

Treatment (mg / kg)	n	Preference for open arm entry	No. of open arm entries	Time spent in open arms	Total no. of entries
Saline, 1 ml, first day	8	50 ± 18.89	3.87 ± 0.54	60.25 ± 11.72	24 ± 1.86
Saline, 1 ml, seventh day	8	50 ± 18.89	3.75 ± 0.49	60.37 ± 9.91	21.25 ± 1.04
Piracetam, 100, first day	6	37.5 ± 18.29	3.0 ± 0.46	74.37 ± 22.13	41.25 ± 4.51
Piracetam, 100, seventh day	6	25 ± 16.36	1.75 ± 0.25	24.37 ± 6.23	19.25 ± 2.01***
PHF, 500, first day	6	50.0 ± 18.89	4.25 ± 0.55	55.37 ± 17.16	31v3.13
PHF, 500, seventh day	6	37.5 ± 18.29	2.25 ± 0.52**	25.12 ± 5.6	14.87 ± 0.95***

Values are each in mean ± S.E.M. *indicates *P* < 0.05,

** indicates *P* < 0.01,

*** indicates *P* < 0.001 when compared to day one values (Two tailed Student’s paired t-test).

Also transfer latency after 24 hours was significantly decreased in the Piracetam-treated group (from 72.66 ± 17.09 to 31.66 ± 4.59, *P* < 0.05) and in PHF-treated group (from 74.66 ± 6.64 to 35.22 ± 5.03, *P* < 0.001) [Table 5].

**Table 5:** Effect on transfer latency as studied on elevated plus maze in rats

Treatment mg / kg	n	Transfer latency in seconds (mean ± SEM)	
		1 hour	24 hours
Saline, 1 ml, first day	8	30.55 ± 6.22	30.22 ± 4.83
Piracetam, 100, first day	6	72.66 ± 17.09	31.66 ± 4.59*
PHF, 500, first day	6	74.66 ± 6.64	35.22 ± 5.03**

Values are each in mean ± S.E.M.

* indicates *P* < 0.05,

** indicates *P* < 0.01, when compared to day one values (Two tailed Student’s paired t-test)

## DISCUSSION

Nootropics represent a new class of psychotropic agents with a selective facilitatory effect on the integrative functions of the central nervous system, particularly on intellectual performance, learning capacity, and memory.[12] A number of drugs like Piracetam and Mentat have now been introduced and used in therapy to ameliorate cognitive deficits.[13] PHF is a polyherbal formulation comprising of selective herbs that have proven memory enhancing action.[14]

Locomotor activity is considered as an index of alertness and a decrease in it is indicative of sedative activity.[15] However, none of the doses of PHF extract were found to have any effect on the locomotor activity. Moreover, the lack of effect on the locomotor activity worked to the advantage of the plant demonstrating nootropic activity. The Elevated Plus Maze (EPM) is a commonly used method to evaluate the anxiety state in animals, however, recent studies of several nootropics and amnesic agents on EPM made this model a widely accepted paradigm to study the learning and memory process in rodents.[16] It is simple, less time consuming, and does not involve the use of any sophisticated equipment or prior training.[17] In the EPM, acquisition (learning) can be considered as transfer latency on the first-day trials and the retention/consolidation (memory) is examined 24 hours later. The animals treated with PHF (500 mg / kg) showed a significant decrease in transfer latency as compared to the control group, which is an indication of the cognitive enhancer effect of PHF in rats.[18] Chronic administration of Piracetam (100 mg / kg) and PHF (500 mg / kg) for seven days reduced the number of open arm entries, time spent in the open arm, and the total number of entries on the seventh day, as compared to the first day treatment.

The passive avoidance test demonstrates the paradigm of short-term memory and is usually supplemented with the elevated plus maze test.[10] PHF produced significant improvement in passive avoidance acquisition and memory retrieval, as compared to the control. The memory improving the effect of PHF manifested as a decrease in latency to reach the Shock Free Zone (SFZ) (acquisition), and a significant decrease in the number of mistakes (descents) the animal made in 15 minutes (retention) on the passive avoidance paradigm. The drug also reduced the latency to reach SFZ after 24 hours. These studies suggested an effectiveness of PHF in improving short-term memory in rats.

Learning the passive avoidance task is known to be processed by the cholinergic synapse.[19] PHF might have produced improvement in passive avoidance acquisition and memory retrieval by the involvement of cholinergic mechanisms or by the GABA receptor inhibition improving short-term memory in rats.[20] Also a modulatory role of the cholinergic or dopaminergic system or 5-HT receptors might be speculated in the efficacious role of PHF in improving short-term memory in the elevated plus maze test.[21]

## CONCLUSION

From the findings of the present study it can be concluded that PHF produces significant improvement in passive avoidance acquisition and memory retrieval in rats. This may be due to the involvement of cholinergic mechanisms or by the GABA receptor inhibition improving short-term memory in rats. These findings have scientifically validated the traditional claim and suggested its valuable role in the treatment of impaired memory functions. The study further reveals that the formulation is devoid of any neurotoxicity or CNS-depressant effect. As the present study is based on the behavioral models without any associated neurochemical estimations, it becomes necessary to carry out specific binding studies and estimations of the neurotransmitter levels in the brain, to understand the exact mechanism of action and extend these results further.
